# Supplementary figures and images for: Molecular assessment of Pocillopora verrucosa (Scleractinia; Pocilloporidae) distribution along a depth gradient in Ludao, Taiwan
Source: PeerJ. 2018 Oct 25;6:e5797. doi: 10.7717/peerj.5797 (PMC6204238; doi:10.7717/peerj.5797)

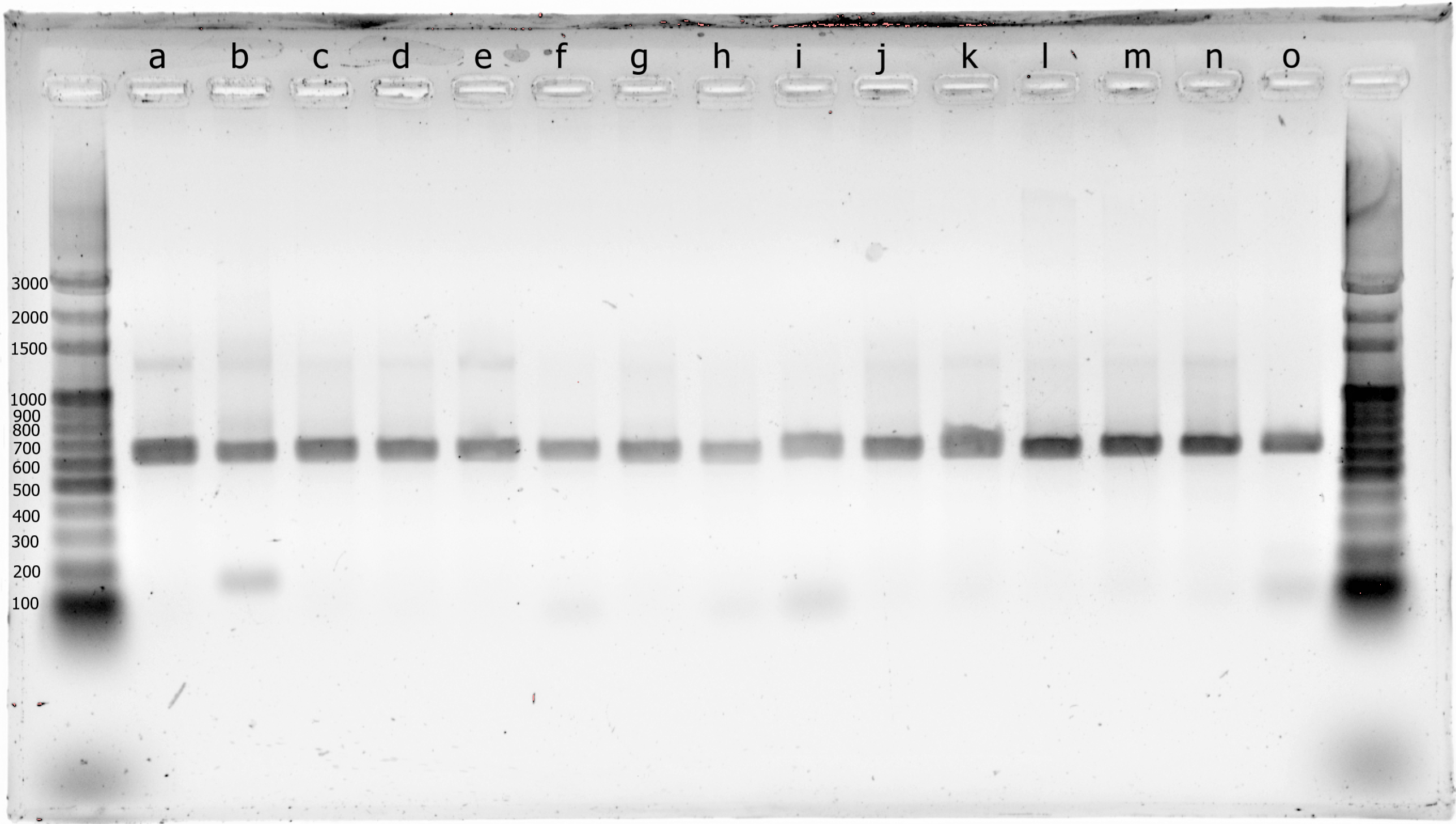

Supplement: Supplemental Information 3 — Lanes 1 and 17 are used for the ladder (bp). Samples are in the following order: a-GI3036, b-GI3037, c-GI3038, d-GI3039, e-GI3040, f-GI3044, g-GI6003, h-GI6004, i-GI6010, j-GI6025, k-GI6056, l-GI6058, m-GI6071, n-GI6075, o-GI6077. [file peerj-06-5797-s003.png]
