# Supplementary material for: Molecular assessment of Pocillopora verrucosa (Scleractinia; Pocilloporidae) distribution along a depth gradient in Ludao, Taiwan
Source: PeerJ. 2018 Oct 25;6:e5797. doi: 10.7717/peerj.5797 (PMC6204238; doi:10.7717/peerj.5797)
Supplement: Supplemental Information 4 — A–H1 (GI3040), B-H2 (GI6058), C- H3 (GI6057), D- H4 (GI6092), E- H5 (GI3014), F- H6 (GI3034), G- H7 (GI6099), H- H8 (GI3018), I- H9 (GI3003). Numbers in brackets are the sample numbers. Scale bar is 6cm long. Photo credit: Stéphane De Palmas. [file peerj-06-5797-s004.pdf]

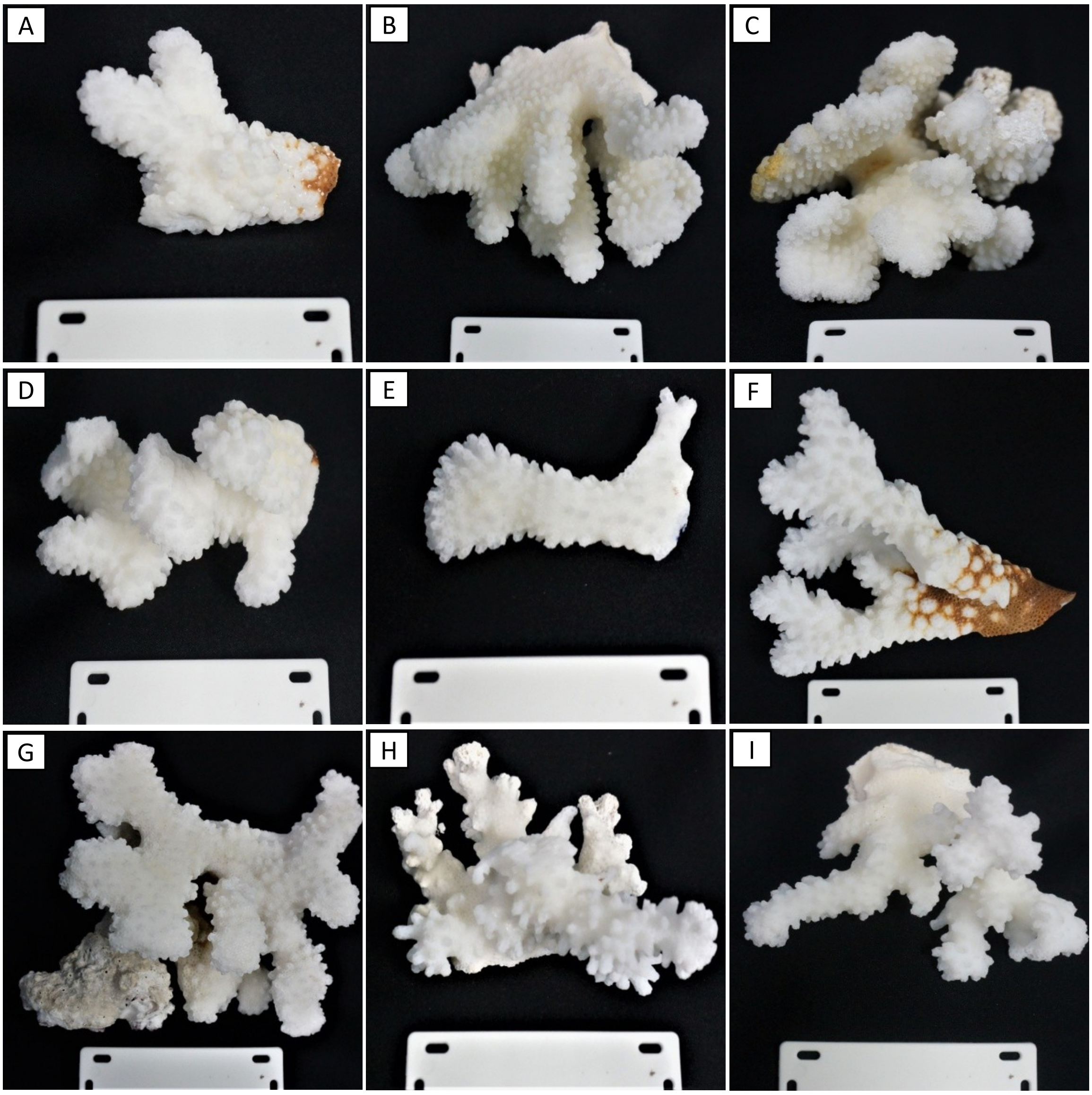

Supplemental Figure S2. Representative coral fragment of each identified haplotypes: A - H1 (GI3040), B-H2 (GI6058), C- H3 (GI6057), D- H4 (GI6092), E- H5 (GI3014), F- H6 (GI3034), G- H7 (GI6099), H- H8 (GI3018), I- H9 (GI3003). Numbers in brackets are the samples numbers. Scale bar is 6cm long.
